# Timing of femoral shaft fracture fixation following major trauma: A retrospective cohort study of United States trauma centers

**DOI:** 10.1371/journal.pmed.1002336

**Published:** 2017-07-05

**Authors:** James P. Byrne, Avery B. Nathens, David Gomez, Daniel Pincus, Richard J. Jenkinson

**Affiliations:** 1 Sunnybrook Research Institute, Sunnybrook Health Sciences Center, Toronto, Ontario, Canada; 2 Clinical Epidemiology Program, Institute of Health Policy, Management and Evaluation, University of Toronto, Toronto, Ontario, Canada; 3 Division of General Surgery, University of Toronto, Toronto, Ontario, Canada; 4 Department of Surgery, Sunnybrook Health Sciences Center, University of Toronto, Toronto, Ontario, Canada; 5 Trauma Quality Improvement Program, American College of Surgeons, Chicago, Illinois, United States of America; 6 Division of Orthopaedic Surgery, University of Toronto, Ontario, Canada; Oregon Health and Science University, UNITED STATES

## Abstract

**Background:**

Femoral shaft fractures are common in major trauma. Early definitive fixation, within 24 hours, is feasible in most patients and is associated with improved outcomes. Nonetheless, variability might exist between trauma centers in timeliness of fixation. Such variability could impact outcomes and would therefore represent a target for quality improvement. We evaluated variability in delayed fixation (≥24 hours) between trauma centers participating in the American College of Surgeons (ACS) Trauma Quality Improvement Program (TQIP) and measured the resultant association with important clinical outcomes at the hospital level.

**Methods and findings:**

A retrospective cohort study was performed using data derived from the ACS TQIP database. Adults with severe injury who underwent definitive fixation of a femoral shaft fracture at a level I or II trauma center participating in ACS TQIP (2012–2015) were included. Patient baseline and injury characteristics that might affect timing of fixation were considered. A hierarchical logistic regression model was used to identify predictors of delayed fixation. Hospital variability in delayed fixation was measured using 2 approaches. First, the random effects output of the hierarchical model was used to identify outlier hospitals where the odds of delayed fixation were significantly higher or lower than average. Second, the median odds ratio (MOR) was calculated to quantify heterogeneity in delayed fixation between hospitals. Finally, complications (pulmonary embolism, deep vein thrombosis, acute respiratory distress syndrome, pneumonia, decubitus ulcer, and death) and hospital length of stay were compared across quartiles of risk-adjusted delayed fixation.

We identified 17,993 patients who underwent definitive fixation at 216 trauma centers. The median injury severity score (ISS) was 13 (interquartile range [IQR] 9–22). Median time to fixation was 15 hours (IQR 7–24 hours) and delayed fixation was performed in 26% of patients. After adjusting for patient characteristics, 57 hospitals (26%) were identified as outliers, reflecting significant practice variation unexplained by patient case mix. The MOR was 1.84, reflecting heterogeneity in delayed fixation across centers. Compared to hospitals in the lowest quartile of delayed fixation, patients treated at hospitals in the highest quartile of delayed fixation suffered 2-fold higher rates of pulmonary embolism (2.6% versus 1.3%; rate ratio [RR] 2.0; 95% CI 1.2–3.2; *P* = 0.005) and required greater length of stay (7 versus 6 days; RR 1.15; 95% CI 1.1–1.19; *P* < 0.001). There was no significant difference with respect to mortality (1.3% versus 0.8%; RR 1.6; 95% CI 1.0–2.8; *P* = 0.066). The main limitations of this study include the inability to classify fractures by severity, challenges related to the heterogeneity of the study population, and the potential for residual confounding due to unmeasured factors.

**Conclusions:**

In this large cohort study of 216 trauma centers, significant practice variability was observed in delayed fixation of femoral shaft fractures, which could not be explained by differences in patient case mix. Patients treated at centers where delayed fixation was most common were at significantly greater risk of pulmonary embolism and required longer hospital stay. Trauma centers should strive to minimize delays in fixation, and quality improvement initiatives should emphasize this recommendation in best practice guidelines.

## Abstract 2

James Byrne and colleagues investigate practice variability for femoral shaft fracture fixation in 216 United States trauma centers.

## Author summary

**Why was this study done?:**

- Femoral shaft fractures are common in patients with blunt multisystem trauma.
- Definitive fixation of femoral shaft fractures is an important process of care at level I and II trauma centers, and the timeliness of fixation might affect center-level outcomes.

**What did the researchers do and find?:**

- Retrospective cohort study of 17,993 patients who underwent definitive fixation of femoral shaft fractures at 216 level I and II trauma centers in the United States
- Hierarchical modelling was used to identify factors associated with delayed fixation (>24 hours). Hospital-level variation in delayed fixation was measured and the association with important clinical outcomes explored.
- Significant variability in delayed fixation was evident between hospitals after adjusting for patient characteristics (range, 0%–82%). One-in-four (*n* = 57) trauma centers were significant outliers, and the median odds ratio was 1.84, indicating significant hospital-level variation in delayed fixation that was not explained by patient case mix.
- Patients treated at hospitals in the highest quartile of delayed fixation were significantly more likely to experience pulmonary embolism (2.6% versus 1.3%; rate ratio [RR] 2.0; 95% CI 1.2–3.2) and required greater length of stay (7 versus 6 days; RR 1.15; 95% CI 1.12–1.19), compared to hospitals in the quartile with the least delayed fixation.

**What do these findings mean?:**

- Significant hospital-level variability in delayed fixation of femur fractures exists, and this appears to affect important clinical outcomes.
- Trauma centers should strive to minimize delayed fixation where possible, and practice management guidelines should emphasize this goal.

## Introduction

Femoral shaft fractures are common in major trauma, often occurring in patients with blunt multiple-system injuries [1]. Early definitive stabilization, within 24 hours, has been associated with decreased risk of thromboembolism, pulmonary complications, and shorter length of stay as compared to delayed fixation [2–4]. While decision-making in patients with severe multiple-system injuries is complex, early definitive care is feasible and safe in the majority of patients [5]. For this reason, surgical fixation within 24 hours is conditionally recommended in current practice management guidelines [1].

Despite recognition that patients with femoral shaft fractures benefit from early fixation, the degree to which this is achieved is unknown. At the patient level, ongoing hemodynamic instability, coagulopathy, and fluctuations in cerebral perfusion [6, 7] are often cited as reasons to postpone definitive fixation. At the hospital level, differences in processes of care predominantly driven by physician decision-making, institutional policies, or allocation of resources are also likely to influence the timing of fixation, independent of patient factors. Taken together, there is potential for variability to exist between hospitals in the number of patients undergoing delayed fixation. Such variability might affect clinical outcomes and would therefore represent an important target for quality improvement.

For this reason, we set out to evaluate the variability in delayed femoral shaft fracture fixation between trauma centers participating in the American College of Surgeons (ACS) Trauma Quality Improvement Program (TQIP) and to determine the resultant association with important clinical outcomes at the hospital level.

## Methods

### Study design

We performed a retrospective cohort study of severely injured patients with femoral shaft fractures who underwent a definitive surgical fixation procedure. The objectives of this study were 3-fold: (1) to determine factors associated with delayed fixation at the patient level, (2) to evaluate variation in delayed fixation between trauma centers, and (3) to assess if delayed fixation is associated with the occurrence of important clinical outcomes at the hospital level. This project was approved by the Sunnybrook Health Sciences Center research ethics board (Toronto, Ontario, Canada). The need for patient informed consent was waived due to the de-identified nature of the data.

### Study population

**Fig 1:**
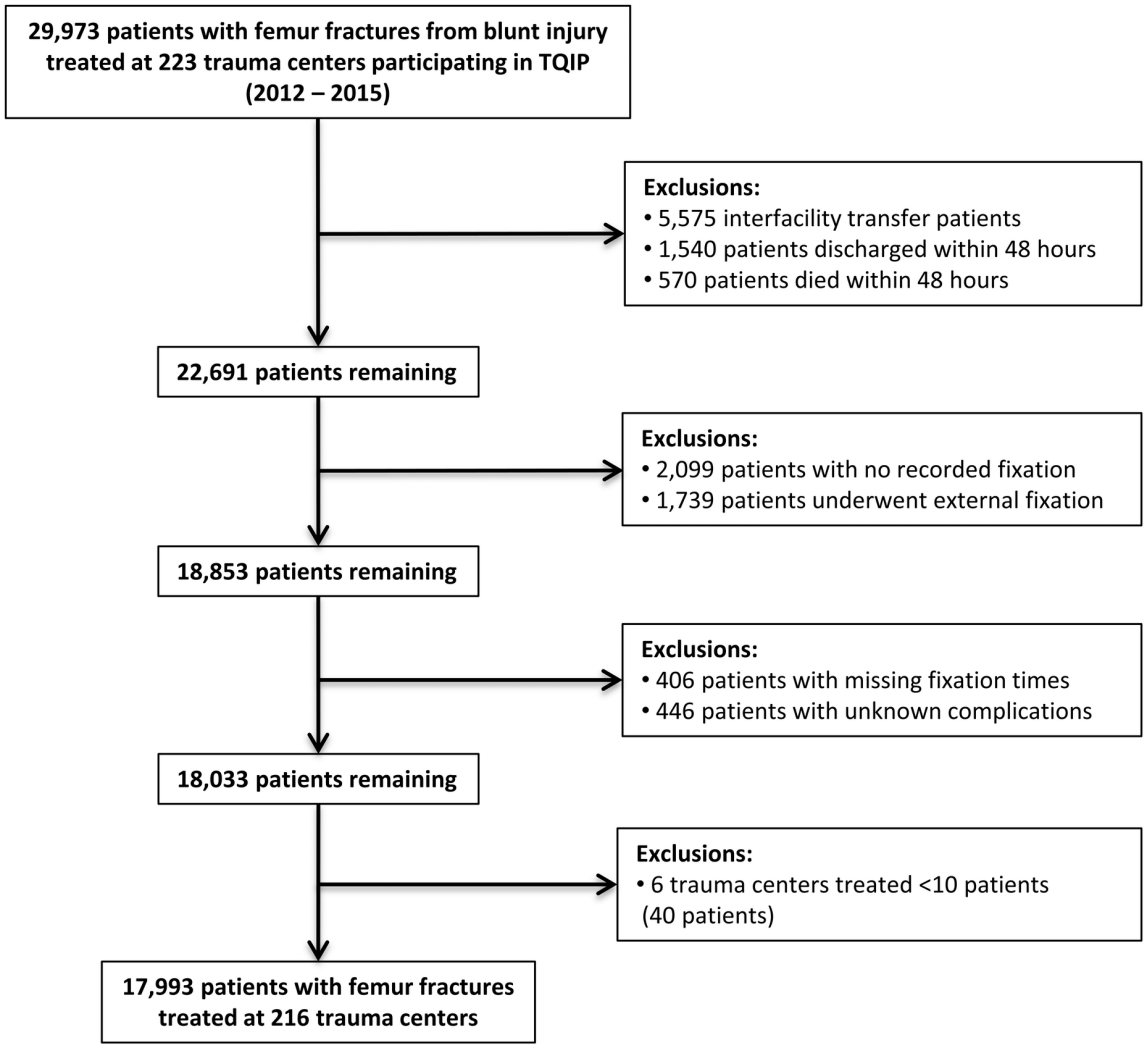
Derivation of the patient cohort. Numbers of patients excluded reflect that patients may have met more than 1 criteria for exclusion.

Data for this study were derived from the ACS TQIP database. Patients treated at level I and II trauma centers participating in ACS TQIP between January 1, 2012 and December 31, 2015 were considered. Derivation of the study cohort is shown in Fig 1. We included all adult patients (≥16 years) with severe injuries (defined as an Abbreviated Injury Score [AIS] ≥ 3 in at least 1 body region) caused by blunt trauma who underwent definitive surgical fixation of a femur fracture. Patients were identified using International Classification of Diseases 9th edition diagnoses codes for open (821.11) or closed (821.01) femoral shaft fractures. While some hospitals began using ICD 10th edition in late 2015, TQIP collected both ICD-9 and -10 during this transitional period, so case ascertainment was not affected for this study. Definitive fixation procedures were identified using ICD-9-CM procedure codes for open reduction with internal fixation (ORIF), closed reduction with internal fixation (CRIF), and internal fixation without fracture reduction (S1 Table). Patients who died or were discharged within 48 hours, underwent interfacility transfer, or did not undergo surgical fixation were excluded.

Patients who underwent external fixation were also excluded, since these often represent patients in physiologic extremis in which definitive fixation is not appropriate. To justify this exclusion, we compared patient baseline and injury characteristics between those who received external versus internal fixation (S2 Table). Patients who received external fixation had patterns of injury that were profoundly more severe than those who received internal fixation, suggesting that external fixation is predominantly performed in a damage control setting [8] and supporting the rationale for exclusion.

### Data source

TQIP was established to provide an opportunity for trauma centers to compare outcomes and processes of care with peer centers [9, 10]. Patients with at least 1 severe injury (AIS ≥ 3 in at least 1 body region) are included. More than 100 patient and hospital variables are collected, including patient baseline characteristics, injury mechanism and severity, emergency department (ED) vital signs, in-hospital procedures, as well as in-hospital outcome information. Reliability of data is ensured through abstractor training and inter-rater reliability audits at participating sites. At the time of this study, there were more than 250 participating ACS or state-verified level I and II trauma centers across North America.

### Study outcome

The primary outcome in this study was delayed fixation of the femur fracture, defined as fixation more than 24 hours after arrival in the ED. This time point was selected to align with current practice management guidelines [1] and evidence for improved clinical outcomes in patients with multiple injuries who undergo fixation within 24 hours [2–5].

### Potential factors influencing timing of fixation

We considered a wide range of patient baseline and injury characteristics that might influence decision-making with respect to the timing of fixation for femur fractures. Patient baseline characteristics included age, sex, race, insurance type, and comorbidities. Injury characteristics included the mechanism of injury, global (injury severity score [ISS]) and anatomic (AIS by body region) injury severity, and open (versus closed) fracture. Specific associated injuries that might influence the timing of fixation included pelvic fractures, tibia or fibula fracture, and spinal cord injury. ED presenting vital signs, early blood transfusion (within 12 hours of arrival), and early surgical interventions (thoracotomy, laparotomy, or neurosurgical intervention within 48 hours) were also considered (S1 Table).

To characterize the hospital environment that might influence the timing of fixation, we classified centers based on trauma center designation level (level I versus II), teaching status (university, community, or nonteaching center), funding type (nonprofit versus for profit), bed size (>600 versus ≤600 beds), and the volume of femoral shaft fractures treated (in quartiles).

### Determining patient factors associated with delayed fixation

At the patient level, standardized differences were used to compare baseline and injury characteristics between patients who underwent delayed versus early fixation [11]. Standardized differences were preferred because standard statistical tests are sensitive to large sample size and would virtually always result in *p* values <0.05 where differences are of no clinical significance. Standardized differences greater than 10% indicated meaningful differences [12].

Patient characteristics were then entered into a hierarchical logistic regression model with delayed fixation as the outcome. This model was a mixed multilevel model that included a random-effects term to account for clustering of patients within trauma centers. The output of this model provided the fixed effects for specific patient characteristics, showing which factors were predictive of delayed fixation.

### Evaluating variability in delayed fixation across trauma centers

Crude rates of delayed fixation were examined across trauma centers. Because differences between hospitals in rates of delayed fixation might be due to differences in patient case mix, we used the multilevel nature of the hierarchical logistic regression model to determine each trauma center’s risk-adjusted tendency for performing delayed fixation. Specifically, the random-effects output from the model provided each trauma center’s unique odds ratio (OR) with 95% confidence interval (CI) for performing delayed fixation, adjusted for patient baseline and injury characteristics.

Variability between trauma centers in the likelihood of performing delayed fixation was quantified first by evaluating hospital outlier status. A hospital where the upper limit of the 95% CI was less than 1 was a low outlier—significantly less likely to perform delayed fixation than the average center. Conversely, if the lower limit of the 95% CI was greater than 1, then the hospital was a high outlier—significantly more likely than average to perform delayed fixation [9, 13]. In this way, the proportion of trauma centers that were outliers provided a measure of significant practice variation not explained by patient case mix.

We also calculated the median odds ratio (MOR) to quantify variability in delayed fixation at the hospital level [14, 15]. In this study, the MOR corresponds to the median value obtained when comparing the adjusted rates of delayed fixation at 2 randomly selected hospitals. It shows the extent to which the individual probability of undergoing delayed fixation is determined by the trauma center at which treatment is received, in comparison to the fixed effects of patient baseline and injury characteristics [16].

### Relationship between hospital tendency for delayed fixation and clinical outcomes

Trauma centers were grouped into quartiles by their unique ORs for delayed fixation. Trauma centers with the lowest ORs were those with the lowest tendency for performing delayed fixation (Quartile 1), while those with the highest ORs were those most likely to perform delayed fixation (Quartile 4). We then compared clinically important outcomes across quartiles. Outcomes included complications such as pulmonary embolism (PE), deep vein thrombosis (DVT), acute respiratory distress syndrome (ARDS), pneumonia, decubitus ulcer, and in-hospital death. Hospital length of stay (in days) was also examined. This approach allowed us to determine if hospital tendency for delayed fixation affects patient outcomes.

### Statistical analysis

Medians and interquartile ranges (IQR) were calculated for continuous variables while relative frequencies were measured for discrete variables. Standardized differences were used to compare patient-level characteristics between groups [11, 12]. Comparisons in hospital-level characteristics were made across quartiles of delayed fixation using the χ^2^ test, with *p* < 0.05 set as the threshold for significance. Rate ratios (RRs) and 95% CIs comparing rates of complications and length of stay were estimated using negative binomial regression.

Variables were selected for inclusion in the hierarchical logistic regression model using a combination of the “change-in-estimate” approach described by Mickey and Greenland [17] and a priori determination of important clinical parameters. The model showed fair discrimination (c statistic, 0.74) and adequate calibration (by the Hosmer—Lemeshow test).

Emergency department vital signs data were missing for less than 4% of patients. Missing values were estimated by a multiple-imputation technique [18]. This approach was preferable to case deletion because of the potential bias associated with nonrandom missing data.

All analyses were performed using SAS software (version 9.4, Cary, NC).

## Results

### Study population

Between 2012 and 2015, the prevalence of femoral shaft fractures among all patients with blunt trauma treated at TQIP centers was 4.8%. We identified 17,993 patients with femoral shaft fractures treated at 216 trauma centers who met inclusion criteria (Fig 1). The median age was 36 years (IQR 23–58 years), and 62% (*n* = 11,183) were male. The majority of patients were injured by motor vehicle crashes (*n* = 7,619; 42%) or falls (*n* = 4,908; 27%) with a median ISS of 13 (IQR 9–22). Open femur fractures were sustained in 12% (*n* = 2,198) of patients. The median time to fracture fixation in all patients was 15 hours (IQR 7–24 hours). Delayed fixation was performed in 26% of patients (*n* = 4,632).

### Patient factors associated with delayed fixation

Table 1 compares baseline and injury characteristics between patients who underwent delayed versus early surgical fixation. Results of the hierarchical logistic model for delayed fixation showed similar results (Table 2). Specifically, increasing age, black race (OR 1.21; 95% CI 1.08–1.35), noncommercial insurance (OR 1.15; 95% CI 1.05–1.26), and comorbidities were significant predictors. Chronic renal failure (OR 2.35; 95% CI 1.55–3.56) and bleeding disorders (including chronic anticoagulation) (OR 1.61; 95% CI 1.36–1.92) were the comorbid conditions most predictive of delayed surgery. Increasing global (ISS) and anatomic injury burden (anatomic AIS), shock or decreased Glasgow coma scale (GCS) in the ED, and need for early surgery were significantly associated with delayed fixation. Conversely, high-energy mechanisms of injury (motor vehicle or motorcycle crash and pedestrian injury) and open femur fractures (OR 0.50; 95% CI 0.44–0.58) were strongly associated with early fixation.

**Table 1:** Patient characteristics associated with delayed fixation.

Parameter	Delayed Fixation (*N* = 4,632)	Early Fixation (*N* = 13,361)	Standardized Difference^a^ (%)
***Baseline Characteristics***			
Median age, years (IQR)	51 (28–71)	32 (23–53)	51.4
Male sex, *n* (%)	2,563 (55.3)	8,620 (64.5)	18.8
Race, *n* (%)			3.2
White	3,160 (68.2)	9,030 (67.6)	
Black	826 (17.8)	2,319 (17.4)	
Other	646 (14.0)	2,012 (15.1)	
Insurance status, *n* (%)			19.1
Commercial	1,234 (26.6)	4,711 (35.3)	
Noncommercial	2,994 (64.6)	7,492 (56.1)	
Other	404 (8.7)	1,158 (8.7)	
Comorbid illness, *n* (%)			
Coronary artery disease	246 (5.3)	226 (1.7)	19.8
Hypertension	1,585 (34.2)	2,511 (18.8)	35.5
Diabetes mellitus	693 (15.0)	922 (6.9)	26.1
Obesity	478 (10.3)	1,149 (8.6)	5.9
Respiratory disease	374 (8.1)	727 (5.4)	10.5
Chronic renal failure	73 (1.6)	44 (0.3)	12.9
Bleeding disorder	361 (7.8)	356 (2.7)	23.2
Functionally dependent	262 (5.7)	224 (1.7)	21.3
***Injury Characteristics***			
Mechanism of injury, *n* (%)			40.5
Fall	1,881 (40.6)	3,027 (22.7)	
Motor vehicle collision	1,634 (35.3)	5,985 (44.8)	
Motorcycle	460 (9.9)	1,953 (14.6)	
Pedestrian	342 (7.4)	1,008 (7.5)	
Other blunt	315 (6.8)	1,388 (10.4)	
Injury severity score, *n* (%)			31.3
9–15	2,625 (56.7)	8,420 (63.0)	
16–25	772 (16.7)	2,917 (21.8)	
26–47	1,090 (23.5)	1,934 (14.5)	
48–75	145 (3.1)	90 (0.7)	
Severe injury AIS ≥ 3, *n* (%)			
Head	757 (16.3)	1,109 (8.3)	24.7
Chest	1,332 (28.8)	2,935 (22.0)	15.7
Abdomen	562 (12.1)	795 (6.0)	21.7
Spine	246 (5.3)	394 (3.0)	11.9
Pelvic fracture, *n* (%)	737 (15.9)	1,650 (12.4)	10.2
Tibia or fibula fracture, *n* (%)	761 (16.4)	2,286 (17.1)	1.8
Spinal cord injury, *n* (%)	94 (2.0)	100 (0.8)	11.0
Open femur fracture, *n* (%)	363 (7.8)	1,835 (13.7)	19.1
***Presenting ED Characteristics***			
Shock in ED^b^, *n* (%)	313 (6.8)	472 (3.5)	14.6
ED GCS motor ≤ 3, *n* (%)	483 (10.4)	527 (3.9)	25.3
Assisted respiration in ED, *n* (%)	499 (10.8)	716 (5.4)	20.0
Early blood transfusion^c^, *n* (%)	883 (19.1)	1,558 (11.7)	20.6
***Early Surgical Intervention***^d^			
Early laparotomy or thoracotomy, *n* (%)	298 (6.4)	255 (1.9)	22.8
Early neurosurgical intervention, *n* (%)	190 (4.1)	99 (0.7)	22.0

^a^ Standardized differences ≥10% represent meaningful differences between groups

^b^ Presenting systolic blood pressure ≤90 mmHg

^c^ Transfusion of packed red blood cells within 12 hours of arrival

^d^ Procedure performed within 48 hours of arrival

AIS, Abbreviated Injury Score; ED, emergency department; GCS, Glasgow coma scale; IQR, interquartile range

**Table 2:** Mixed multilevel model for delayed fixation.

Parameter	Odds of Delayed Fixation	95% CI
***Baseline Characteristics***		
Age, years (linear)	1.02	1.02–1.02
Male sex	1.03	0.95–1.12
Race		
White	Reference	
Black	1.21	1.08–1.35
Other	0.96	0.85–1.08
Insurance status		
Commercial	Reference	
Noncommercial	1.15	1.05–1.26
Other	1.29	1.09–1.52
Comorbid illness		
Coronary artery disease	1.58	1.28–1.95
Hypertension	1.09	0.98–1.21
Diabetes mellitus	1.37	1.20–1.55
Obesity	1.16	1.02–1.33
Respiratory disease	1.13	0.97–1.31
Chronic renal failure	2.35	1.55–3.56
Bleeding disorder	1.61	1.36–1.92
Functionally dependent	1.55	1.26–1.91
***Injury Characteristics***		
Mechanism of injury		
Fall	Reference	
Motor vehicle collision	0.61	0.54–0.69
Motorcycle	0.58	0.50–0.68
Pedestrian	0.59	0.50–0.70
Other blunt	0.63	0.53–0.74
Injury severity score		
9–15	Reference	
16–25	1.20	1.06–1.38
26–47	1.67	1.35–2.06
48–75	2.24	1.49–3.35
Severe injury AIS ≥ 3		
Head	1.44	1.23–1.68
Chest	1.15	1.00–1.32
Abdomen	1.58	1.34–1.86
Spine	1.16	0.93–1.45
Pelvic fracture	1.12	1.00–1.26
Tibia or fibula fracture	1.03	0.93–1.15
Spinal cord injury	1.61	1.12–2.32
Open femur fracture	0.50	0.44–0.58
***Presenting ED Characteristics***		
Shock in ED	1.25	1.04–1.49
ED GCS motor ≤ 3	1.76	1.42–2.17
Assisted respiration in ED	1.12	0.92–1.35
Early blood transfusion	1.29	1.14–1.45
***Early Surgical Intervention (<48 hours)***		
Early laparotomy or thoracotomy	3.19	2.36–4.31
Early neurosurgical intervention	2.23	1.79–2.77

ORs and 95% CIs estimated using hierarchical model accounting clustering of patients within centers

c statistic, 0.74

AIS, Abbreviated Injury Score; CI, confidence interval; ED, emergency department; GCS, Glasgow coma scale; OR, odds ratio

### Variability in delayed fixation across trauma centers

Across the 216 trauma centers included in this study, the proportion of patients who underwent delayed fixation ranged from 0% to 82% (median 26%; IQR 19%–35%).

**Fig 2:**
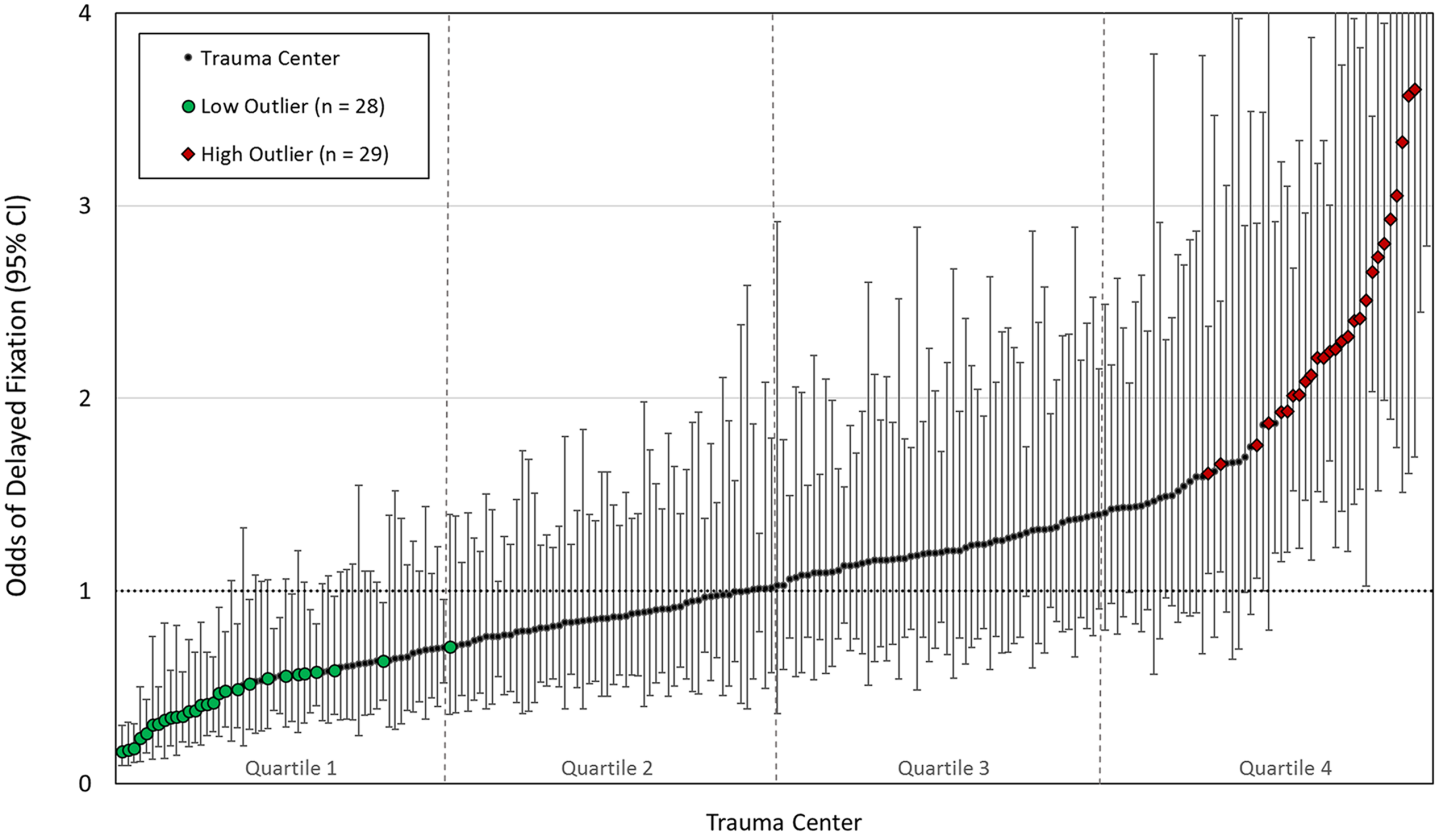
Caterpillar plot showing trauma center odds ratios (ORs) and 95% confidence intervals (Cis) for delayed fixation, risk-adjusted for patient baseline and injury characteristics. High outliers (greater than average odds of delayed fixation) and low outliers (lower than average odds of delayed fixation) are shown. In total, 57 (26%) centers showed significant differences in rates of delayed fixation unexplained by patient case mix. Data available in S3 Table.

Each trauma center’s unique risk-adjusted OR and 95% CI for performing delayed fixation was estimated using the random-effects output of the hierarchical logistic regression model. The results of this analysis are shown in Fig 2. Based on these results, 29 trauma centers (13%) were found to be high outliers (significantly more likely than average to perform delayed fixation). Conversely, 28 trauma centers (13%) were low outliers (significantly less likely than average to perform delayed fixation). Taken together, 1 in 4 trauma centers (*n* = 57; 26%) showed significant differences in the likelihood of performing delayed fixation not attributable to patient case mix.

The MOR for delayed fixation across trauma centers was 1.84. In other words, if 2 hospitals were randomly selected from our cohort, the median increase in the odds of delayed fixation incurred by moving from the lower to higher risk hospital was 84%. This value indicates that the individual probability of receiving delayed fixation is dictated by the trauma center at which treatment is received to a meaningful extent and reflects a large degree of heterogeneity between hospitals in rates of delayed fixation that are unexplained by patient factors.

### Influence of hospital tendency for delayed fixation on clinical outcomes

Trauma centers were grouped into quartiles by their risk-adjusted ORs for delayed fixation (Fig 2). Compared to hospitals where delayed fixation was minimized (Quartile 1), patients treated at hospitals in the highest quartile (Quartile 4) were 4 times more likely to undergo delayed fixation (OR 4.3; 95% CI 3.9–4.8), despite minimal differences in patient and hospital characteristics across quartiles (Tables 3 and 4).

**Table 3:** Patient characteristics by hospital quartile of delayed fixation.

Parameter	Trauma Center Tendency for Delayed Fixation				Standardized Difference^a^ (%)
	Least Delayed Fixation		Most Delayed Fixation		
	Quartile 1 (*N* = 4,779)	Quartile 2 (*N* = 4,897)	Quartile 3 (*N* = 4,293)	Quartile 4 (*N* = 4,024)	
Median rate of delayed fixation, % (IQR)	14 (10–18)	23 (20–26)	31 (26–34)	41 (35–47)	
Odds of delayed fixation (95% CI)	Reference	1.8 (1.7–2.1)	2.7 (2.4–3.0)	4.3 (3.9–4.8)	
***Baseline Characteristics***					
Median age, years (IQR)	36 (24–59)	34 (23–56)	37 (24–60)	35 (23–57)	3.7
Male sex, *n* (%)	2,981 (62.4)	3,034 (62.0)	2,614 (60.9)	2,554 (63.5)	2.3
Race, *n* (%)					26.2
White	3,547 (74.2)	3,312 (67.6)	2,808 (65.4)	2,523 (62.7)	
Black	504 (10.6)	947 (19.3)	973 (22.7)	721 (17.9)	
Other	728 (15.2)	638 (13.0)	512 (11.9)	780 (19.4)	
Insurance status, *n* (%)					5.2
Commercial	1,582 (33.1)	1,705 (34.8)	1,409 (32.8)	1,249 (31.0)	
Noncommercial	2,752 (57.6)	2,817 (57.5)	2,561 (60.0)	2,356 (58.6)	
Other	445 (9.3)	375 (7.7)	323 (7.5)	419 (10.4)	
Comorbid illness, *n* (%)					
Coronary artery disease	162 (3.4)	120 (2.5)	111 (2.6)	79 (2.0)	8.9
Hypertension	1,085 (22.7)	1,071 (21.9)	1,055 (24.6)	885 (22.0)	1.7
Diabetes mellitus	441 (9.2)	434 (8.9)	386 (9.0)	354 (8.8)	1.5
Obesity	409 (8.6)	527 (10.8)	337 (7.9)	354 (8.0)	0.9
Respiratory disease	269 (5.6)	315 (6.4)	273 (6.4)	244 (6.1)	1.9
Chronic renal failure	33 (0.7)	30 (0.6)	37 (0.9)	17 (0.4)	3.6
Bleeding disorder	224 (4.7)	193 (3.9)	168 (3.9)	132 (3.3)	7.2
Functionally dependent	96 (2.0)	140 (2.9)	133 (3.1)	117 (2.9)	5.8
***Injury Characteristics***					
Mechanism of injury, *n* (%)					3.6
Fall	1,302 (27.2)	1,200 (24.5)	1,297 (30.2)	1,109 (27.6)	
Motor vehicle collision	1,980 (41.4)	2,269 (46.3)	1,709 (39.8)	1,661 (41.3)	
Motorcycle	668 (14.0)	642 (13.1)	567 (13.2)	536 (13.3)	
Pedestrian	342 (7.2)	345 (7.1)	343 (8.0)	320 (8.0)	
Other blunt	487 (10.2)	441 (9.0)	377 (8.8)	398 (9.9)	
Injury severity score, *n* (%)					5.4
9–15	2,879 (60.2)	2,870 (58.6)	2,771 (64.6)	2,525 (62.8)	
16–25	999 (20.9)	1,056 (21.6)	829 (19.3)	805 (20.0)	
26–47	837 (17.5)	907 (18.5)	639 (14.9)	641 (15.9)	
48–75	64 (1.3)	64 (1.3)	54 (1.3)	53 (1.3)	
Severe injury AIS ≥ 3, *n* (%)					
Head	517 (10.8)	502 (10.3)	418 (9.7)	429 (10.7)	0.5
Chest	1,172 (24.5)	1,285 (26.2)	887 (20.7)	923 (22.9)	3.7
Abdomen	386 (8.1)	383 (7.8)	284 (6.6)	304 (7.6)	2.0
Spine	151 (3.2)	204 (4.2)	152 (3.5)	133 (3.3)	0.8
Pelvic fracture, *n* (%)	607 (12.7)	681 (13.9)	545 (12.7)	554 (13.8)	3.2
Tibia or fibula fracture, *n* (%)	857 (17.9)	879 (18.0)	662 (15.4)	649 (16.1)	4.8
Spinal cord injury, *n* (%)	39 (0.8)	61 (1.3)	56 (1.3)	38 (0.9)	1.4
Open femur fracture, *n* (%)	607 (12.7)	642 (13.1)	491 (11.4)	458 (11.4)	4.1
***Presenting ED Characteristics***					
Shock in ED^b^, *n* (%)	216 (4.5)	242 (4.9)	152 (3.5)	175 (4.4)	0.8
ED GCS motor ≤ 3, *n* (%)	250 (5.2)	297 (6.1)	252 (5.9)	211 (5.2)	0.1
Assisted respiration in ED, *n* (%)	264 (5.5)	318 (6.5)	317 (7.4)	316 (7.9)	9.3
Early blood transfusion^c^, *n* (%)	647 (13.5)	743 (15.2)	593 (13.8)	458 (11.4)	6.5
***Early Surgical Intervention***^d^					
Early laparotomy or thoracotomy, *n* (%)	166 (3.5)	137 (2.8)	120 (2.8)	130 (3.2)	1.4
Early neurosurgical intervention, *n* (%)	88 (1.8)	75 (1.5)	56 (1.3)	70 (1.7)	0.8

^a^ Standardized differences ≥10% represent meaningful differences between groups (calculated based on comparison of Quartiles 1 and 4)

^b^ Presenting systolic blood pressure ≤ 90 mmHg

^c^ Transfusion of packed red blood cells within 12 hours of arrival

^d^ Procedure performed within 48 hours of arrival

AIS, Abbreviated Injury Score; CI, confidence interval; ED, emergency department; GCS, Glasgow coma scale; IQR, interquartile range

**Table 4:** Trauma center characteristics by hospital quartile of delayed fixation.

Parameter	Trauma Center Tendency for Delayed Fixation				*P* value
	Least Delayed Fixation		Most Delayed Fixation		
	Quartile 1 (*N* = 54)	Quartile 2 (*N* = 54)	Quartile 3 (*N* = 54)	Quartile 4 (*N* = 54)	
Median rate of delayed fixation, % (IQR)	14 (10–18)	23 (20–26)	31 (26–34)	41 (35–47)	
Odds of delayed fixation (95% CI)	Reference	1.8 (1.7–2.1)	2.7 (2.4–3.0)	4.3 (3.9–4.8)	
*Level of designation*, *n (%)*					
Level I (versus Level II)	32 (59.3)	29 (53.7)	34 (63.0)	40 (74.1)	0.164
*Teaching status*, *n (%)*					0.095
University	18 (33.3)	23 (42.6)	32 (59.3)	28 (51.9)	
Community	30 (55.6)	23 (42.6)	15 (27.8)	22 (40.7)	
Nonteaching	6 (11.1)	8 (14.8)	7 (13.0)	4 (7.4)	
*Hospital funding type*, *n (%)*					
Nonprofit (versus For profit)	50 (92.6)	47 (87.0)	46 (85.2)	49 (90.7)	0.599
*Bed size*, *n (%)*					
>600 beds (versus ≤600 beds)	20 (37.0)	23 (42.6)	20 (37.0)	20 (37.0)	0.913
*Femur facture volume*, *n (%)*					0.682
Quartile 1 (<41 patients)	8 (14.8)	15 (27.8)	14 (25.9)	14 (25.9)	
Quartile 2 (41–64 patients)	14 (25.9)	12 (22.2)	14 (25.9)	17 (31.5)	
Quartile 3 (65–109 patients)	14 (25.9)	14 (25.9)	12 (22.2)	14 (25.9)	
Quartile 4 (>109 patients)	18 (33.3)	13 (24.1)	14 (25.9)	9 (16.7)	

CI, confidence interval; IQR, interquartile range

**Fig 3:**
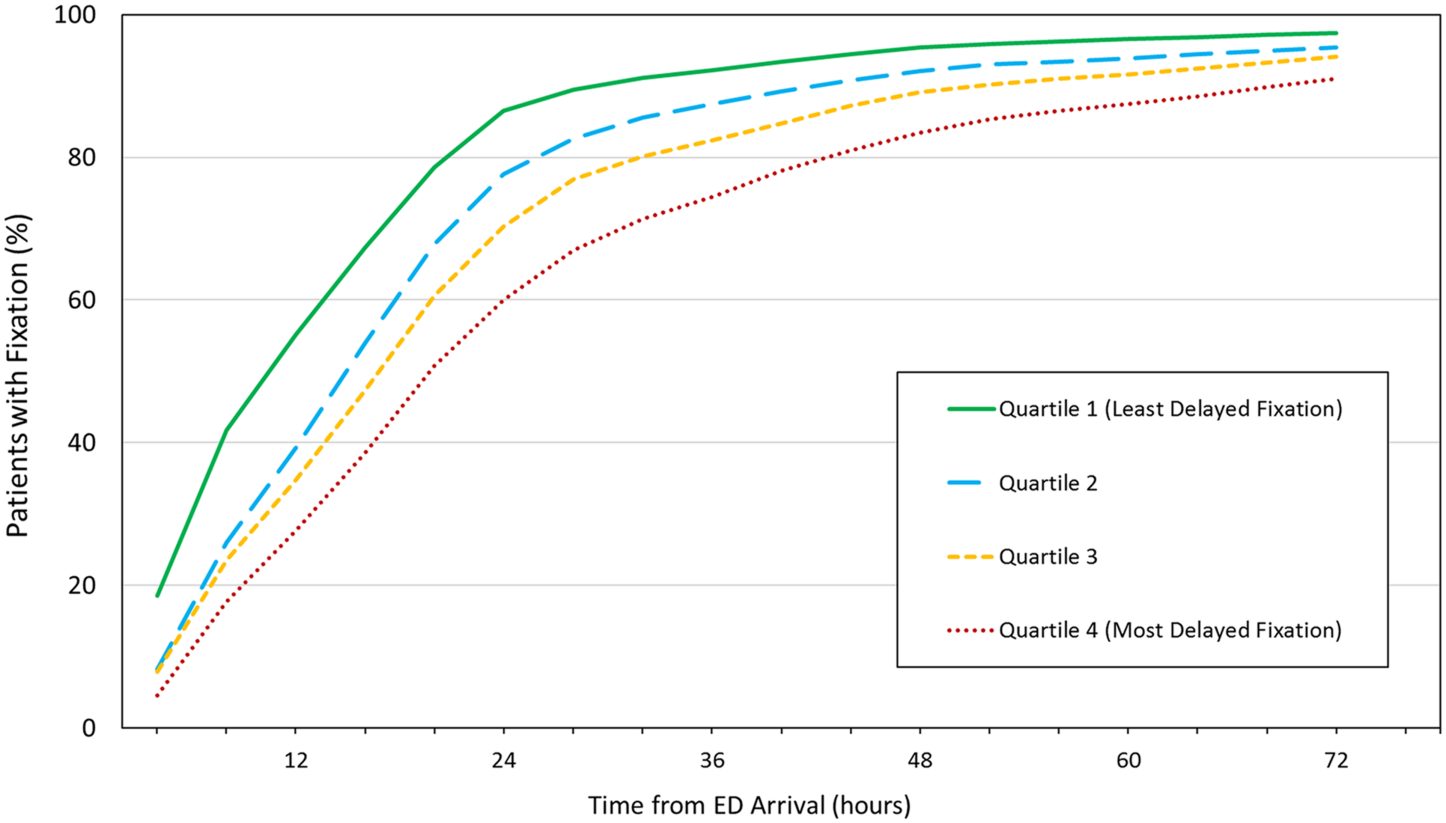
Cumulative percentage of patients receiving definitive fixation as a function of time from emergency department (ED) arrival. Hospitals in the highest quartile of delayed fixation (Quartile 4) achieved fixation in only 60% of patients at 24 hours, whereas 87% of patients underwent fixation within 24 hours at hospitals where delays were minimized (Quartile 1). Data available in S4 Table.

Fig 3 compares the timing of surgical fixation across quartiles. Despite negligible differences in patient case mix, hospitals in the highest quartile of delayed fixation achieved fixation in only 60% of patients at 24 hours (2,410 of 4,024 patients), compared to 87% at hospitals where delayed fixation was least common (4,133 of 4,779 patients).

Finally, clinical outcomes were compared across quartiles (Table 5). Patients treated at trauma centers in the highest quartile of delayed fixation were at significantly higher risk of PE (mean PE rate 2.6% versus 1.3%; RR 2.0; 95% CI 1.2–3.2). A similar relationship was not identified with other complications. Trauma centers most likely to perform delayed fixation also had significantly longer lengths of stay (median 7 days versus 6 days; RR 1.15; 95% CI 1.12–1.19).

**Table 5:** Outcomes by hospital quartile for delayed fixation.

Parameter	Least Delayed Fixation		Most Delayed Fixation		*P* value
	Quartile 1 (*N* = 54)	Quartile 2 (*N* = 54)	Quartile 3 (*N* = 54)	Quartile 4 (*N* = 54)	
Median rate of delayed fixation, % (IQR)	14 (10–18)	23 (20–26)	31 (26–34)	41 (35–47)	
Odds of delayed fixation (95% CI)	Reference	1.8 (1.7–2.1)	2.7 (2.4–3.0)	4.3 (3.9–4.8)	
Pulmonary embolism					
Mean rate (±SD)	1.3 (1.4)	1.6 (2.1)	1.7 (2.0)	2.6 (3.7)	0.005
Rate ratio (95% CI)	Reference	1.2 (0.7–2.0)	1.3 (0.8–2.2)	2.0 (1.2–3.2)	
Deep vein thrombosis					
Mean rate (±SD)	3.1 (2.9)	1.7 (1.9)	2.8 (3.3)	2.8 (3.2)	0.573
Rate ratio (95% CI)	Reference	0.5 (0.4–0.8)	0.9 (0.6–1.3)	0.9 (0.6–1.3)	
Acute Respiratory Distress Syndrome					
Mean rate (±SD)	1.2 (1.8)	1.7 (2.9)	1.1 (1.8)	2.1 (3.4)	0.054
Rate ratio (95% CI)	Reference	1.5 (0.8–2.7)	1.0 (0.5–1.8)	1.8 (1.0–3.2)	
Pneumonia					
Mean rate (±SD)	3.6 (2.8)	3.6 (2.8)	3.7 (4.1)	4.8 (4.4)	0.112
Rate ratio (95% CI)	Reference	1.0 (0.7–1.4)	1.0 (0.7–1.5)	1.3 (0.9–1.9)	
Decubitus ulcer					
Mean rate (±SD)	1.4 (2.2)	1.1 (1.5)	1.1 (1.6)	1.1 (1.4)	0.402
Rate ratio (95% CI)	Reference	0.8 (0.5–1.4)	0.8 (0.5–1.3)	0.8 (0.5–1.4)	
Death					
Mean rate (±SD)	0.8 (1.1)	1.3 (1.5)	1.6 (2.5)	1.3 (1.5)	0.066
Rate ratio (95% CI)	Reference	1.6 (0.9–2.7)	2.0 (1.2–3.3)	1.6 (1.0–2.8)	
Hospital length of stay					
Median days (IQR)	6 (5–10)	7 (5–11)	7 (5–11)	7 (5–12)	<0.001
Rate ratio (95% CI)	Reference	1.06 (1.03–1.09)	1.10 (1.07–1.13)	1.15 (1.12–1.19)	

CI, confidence interval; IQR, interquartile range

## Discussion

In this retrospective study of patients with femoral shaft fractures, we found significant variability between trauma centers in rates of delayed surgical fixation. This variability did not appear to be explained by differences in patient case mix between hospitals, indicating that differences in processes of care might exist that influence the timing of fixation. These differences were associated with patient outcomes, with significantly higher observed rates of PE and greater lengths of stay at trauma centers with the greatest tendency for performing delayed fixation.

Trauma centers must meet standardized criteria and undergo a rigorous process for external verification to maintain designation [19]. This process is designed to ensure consistency with respect to the resources and quality of care injured patient receive. Therefore, in theory, the management of patients with multiple injuries should be comparable at all designated trauma centers. Nonetheless, it is recognized that variability in patient outcomes persist [9, 10]. In response to this recognition, the ACS established TQIP, a quality improvement initiative with the goal of identifying structures and processes of care that distinguish high-performing centers. The management of femoral shaft fractures, a common clinical entity, is one such process with the potential to influence important clinical outcomes.

While evidence is strong that definitive fixation within 24 hours is both feasible [5] and associated with improved outcomes [2–4], managing the therapeutic needs of patients with multiple injuries remains challenging. In our cohort, increasing age and greater comorbidity were significantly associated with delayed fixation. Chronic renal failure and bleeding disorders (which include chronic anticoagulation) were the comorbid illnesses most predictive of delayed surgery. These findings represent the strong influence of comorbidity on clinical decision-making in this patient population and the additional time required to coordinate surgical treatment in medically-complex patients. Severe head injury, spinal cord injury, decreased GCS and early neurosurgery were also significant predictors of delayed fixation. These observations highlight common clinical scenarios in which orthopedic interventions are often deferred in patients with major injury. Fluctuations in cerebral perfusion could cause harm by inflicting secondary injury and are therefore avoided in patients with neurotrauma [7, 20, 21]. Therefore, supportive management in an intensive care setting is often the initial priority in such patients. Hemorrhage control and correction of coagulopathy are other common reasons for postponing the treatment of orthopedic injuries, with the goal of minimizing occult hypoperfusion [6]. Our findings that severe abdominal injury, shock in the ED, early transfusion, and early thoracic or abdominal surgery were predictors of delayed fixation are concordant with this practice.

After adjusting for the patient baseline and injury characteristics discussed, significant variability remained between trauma centers in risk-adjusted odds of delayed fixation. One in four trauma centers were outliers, significantly more or less likely to perform delayed fixation, and the MOR between hospitals was 1.84. These findings indicate that a large degree of variability in the management of femoral shaft fractures exists between trauma centers, not explained by differences in patient case mix. This hospital-level variability is likely to represent differences in processes of care that affect the timing of fixation, such as physician decision-making and preferences, institutional policies, or allocation of resources. This interpretation is further supported by the observation that surgical fixation occurred more rapidly at trauma centers in the lowest quartile of delayed fixation, despite minimal differences in patient or hospital characteristics across quartiles.

Patients treated at trauma centers with the greatest tendency for delayed fixation suffered rates of PE 2-fold higher than at centers where delayed fixation was minimized. PE is an important cause of mortality following major trauma [22]. Patients with severe lower extremity injuries are at high risk [23]. We recently found that femoral shaft fracture was a strong predictor of PE in a mixed trauma population [24]. Many PE in patients with femur fractures occur within 72 hours of injury [25], likely reflecting the synergistic effect of acute tissue trauma and venous stasis in the lower extremity on formation of early thromboembolism. Early definitive fixation has been shown to reduce the risk of PE [2, 4]. Our findings suggest that adopting a hospital policy to minimize the proportion of patients who undergo delayed fixation could be an actionable quality-improvement strategy for reducing the risk of PE in this patient population.

Interestingly, we did not find a significant relationship between other complications and hospital tendency for delayed fixation. This is likely due to thorough risk-adjustment that effectively minimized differences in patient case mix most related to risk for DVT, ARDS, pneumonia, pressure ulcers, or mortality. PE frequently occur in trauma patients without demonstrated DVT [26], and hospital rates of DVT are influenced by institutional screening protocols [27]. Taken together, our results suggest that patient injury factors likely explain much of the variability between hospitals in the occurrence of these complications, more so than processes of care related to timing of fixation.

Our work points to actionable strategies for trauma center quality improvement. First, as stated, hospitals should consider the timeliness with which femoral shaft fractures are stabilized within their center. Our findings suggest that minimizing delays in fixation beyond 24 hours, where clinically acceptable, may lead to fewer PE and earlier patient discharge. Second, to support these efforts, broader quality-improvement initiatives should consider including time to definitive fixation, or the proportion of patients receiving fixation within 24 hours, in benchmarking reports. TQIP provides these data to participating centers, empowering them to set quality-improvement targets. Finally, best practice guidelines [1, 28] should consider including a recommendation for achieving definitive fixation within 24 hours, acknowledging that clinical considerations may supersede this recommendation.

This study has several potential limitations. First, the trauma centers included in this study were level I and II centers; therefore, our findings may not be generalizable to all hospitals in which femoral shaft fractures are treated. However, our cohort of 216 hospitals does capture the clinical environment in which the majority of patients with major trauma should receive treatment within an established trauma system. Second, the TQIP database does not capture traditional fracture severity grading systems (such as the Gustilo-Anderson classification) that could influence timing of fixation. However, we did consider open versus closed fracture status to overcome this limitation. Finally, as in all adjusted analyses, there is potential for residual confounding due to unmeasured factors. Perhaps the greatest source for residual confounding in this study is related to the heterogeneity of the study population. Specifically, the clinical considerations for management of patients with isolated femur fractures are different from those with multiple severe injuries. However, by considering anatomic injury severity, specific patterns of injury, ED vital signs, as well as early interventions such as transfusion and surgery, we were able to account for the majority of factors likely to influence physician decision-making related to the treatment of femoral shaft fractures.

Acknowledging these limitations, the results of this study suggest that significant variability persists between trauma centers in delayed fixation of femoral shaft fractures, independent of patient case mix. Patients treated at centers in which delayed fixation is most common appear to be at greater risk of PE and require longer hospital stay. Trauma centers should strive to achieve definitive fixation within 24 hours and quality-improvement initiatives should emphasize this recommendation in best practice guidelines.

## Conclusion

In this large cohort study of 216 trauma centers, significant variability in delayed fixation of femoral shaft fractures was observed between hospitals that was not explained by patient case mix. Patients treated at centers in which delayed fixation was most common were at significantly greater risk of PE and required longer hospital stay. Trauma centers should strive to minimize delays in fixation and quality-improvement initiatives should emphasize this recommendation in best practice guidelines.

## Supporting information

S1 Table ICD-9-CM procedure codes used for ascertainment of interventions. (DOCX)

S2 Table Comparison of patients receiving external versus internal fixation. (DOCX)

S3 Table Point estimates and 95% confidence intervals of center-level random effects for delayed fixation. (DOCX)

S4 Table Cumulative proportion of patients with fixation over time from ED arrival. (DOCX)

S1 STROBE Checklist (DOC)

S1 Study Protocol (DOCX)

## Abbreviations

ACS: American College of Surgeons
AIS: Abbreviated Injury Score
ARDS: acute respiratory distress syndrome
CI: confidence interval
DVT: deep vein thrombosis
ED: emergency department
GCS: Glasgow coma scale
IQR: interquartile range
ISS: injury severity score
MOR: median odds ratio
OR: odds ratio
PE: pulmonary embolism
RR: rate ratio
TQIP: Trauma Quality Improvement Program
