# Supplementary material for: Timing of femoral shaft fracture fixation following major trauma: A retrospective cohort study of United States trauma centers
Source: PLoS Med. 2017 Jul 5;14(7):e1002336. doi: 10.1371/journal.pmed.1002336 (PMC5497944; doi:10.1371/journal.pmed.1002336)
Supplement: S1 Table — (DOCX) [file pmed.1002336.s001.docx]

| **Supplementary Table 1.** ICD-9-CM procedure codes used for ascertainment of interventions | |
| --- | --- |
| **Femur Fixation Procedures** | **ICD-9-CM procedure code** |
| Open reduction of fracture with internal fixation | 79.35 |
| Closed reduction of fracture with internal fixation | 79.15 |
| Internal fixation of bone without fracture reduction | 78.55 |
| **Other Interventions** | **ICD-9-CM procedure code** |
| Transfusion of packed red blood cells | 99.04 |
| Craniotomy, craniectomy | 01.24, 01.25 |
| Intracranial monitor or drain | 01.10, 01.16, 01.17, 02.21, 02.22 |
| Thoracotomy | 34.02 |
| Laparotomy | 54.11 |
| ICD, International Classification of Diseases | |
