# Supplementary material for: Timing of femoral shaft fracture fixation following major trauma: A retrospective cohort study of United States trauma centers
Source: PLoS Med. 2017 Jul 5;14(7):e1002336. doi: 10.1371/journal.pmed.1002336 (PMC5497944; doi:10.1371/journal.pmed.1002336)
Supplement: S2 Table — (DOCX) [file pmed.1002336.s002.docx]

| **Supplementary Table 2.** Comparison of patients receiving external versus internal fixation | | | |
| --- | --- | --- | --- |
| Parameter | External Fixation  (N = 1,739) | Internal Fixation  (N = 17,993) | Standardized Difference^a^ (%) |
| ***Baseline Characteristics*** |  |  |  |
| Median age, years (IQR) | 34 (24 – 50) | 36 (23 – 58) | 14.9 |
| Male sex, n (%) | 1,216 (69.9) | 11,183 (62.2) | 16.5 |
| Race, n (%) |  |  | 9.0 |
| White | 1,186 (68.2) | 12,190 (67.8) |  |
| Black | 343 (19.7) | 3,145 (17.5) |  |
| Other | 210 (12.1) | 2,658 (14.8) |  |
| Insurance status, n (%) |  |  | 11.0 |
| Commercial | 655 (37.7) | 5,945 (33.0) |  |
| Non-commercial | 966 (55.6) | 10,486 (58.3) |  |
| Other | 118 (6.8) | 1,562 (8.7) |  |
| Comorbid illness, n (%) |  |  |  |
| Coronary artery disease | 20 (1.2) | 472 (2.6) | 10.8 |
| Hypertension | 277 (15.9) | 4,096 (22.8) | 17.4 |
| Diabetes mellitus | 118 (6.8) | 1,615 (9.0) | 8.1 |
| Obesity | 203 (11.7) | 1,627 (9.0) | 8.6 |
| Respiratory disease | 96 (5.5) | 1,101 (6.1) | 2.6 |
| Chronic renal failure | 6 (0.4) | 117 (0.7) | 4.3 |
| Bleeding disorder | 54 (3.1) | 717 (4.0) | 4.8 |
| Functionally dependent | 10 (0.6) | 486 (2.7) | 16.8 |
|  |  |  |  |
| ***Injury Characteristics*** |  |  |  |
| Mechanism of injury, n (%) |  |  | 63.0 |
| Fall | 104 (6.0) | 4,908 (27.3) |  |
| Motor vehicle collision | 914 (52.6) | 7,619 (42.3) |  |
| Motorcycle | 411 (23.6) | 2,413 (13.4) |  |
| Pedestrian | 174 (10.0) | 1,350 (7.5) |  |
| Other blunt | 136 (7.8) | 1,703 (9.5) |  |
| Injury severity score, n (%) |  |  | 73.9 |
| 9 – 15 | 512 (29.4) | 11,045 (61.4) |  |
| 16 – 25 | 419 (24.1) | 3,689 (20.5) |  |
| 26 – 47 | 730 (42.0) | 3,024 (16.8) |  |
| 48 – 75 | 78 (4.5) | 235 (1.3) |  |
| Severe injury AIS ≥ 3, n (%) |  |  |  |
| Head | 421 (24.2) | 1,866 (10.4) | 37.2 |
| Chest | 856 (49.2) | 4,267 (23.7) | 55.0 |
| Abdomen | 359 (20.6) | 1,357 (7.5) | 38.3 |
| Spine | 142 (8.2) | 640 (3.6) | 19.7 |
| Pelvic fracture, n (%) | 473 (27.2) | 2,387 (13.3) | 35.2 |
| Tibia or fibula fracture, n (%) | 820 (47.2) | 3,047 (16.9) | 68.5 |
| Spinal cord injury, n (%) | 42 (2.4) | 194 (1.1) | 10.2 |
|  |  |  |  |
| Open femur fracture, n (%) | 667 (38.4) | 2,198 (12.2) | 63.1 |
|  |  |  |  |
| ***Presenting ED Characteristics*** |  |  |  |
| Shock in ED^b^, n (%) | 239 (13.7) | 785 (4.4) | 33.1 |
| ED GCS motor ≤ 3, n (%) | 331 (19.0) | 1,010 (5.6) | 41.7 |
| Assisted respiration in ED, n (%) | 309 (17.8) | 1,215 (6.8) | 34.1 |
| Early blood transfusion^c^, n (%) | 717 (41.2) | 2,441 (13.6) | 65.2 |
|  |  |  |  |
| ***Early Surgical Intervention^d^*** |  |  |  |
| Early laparotomy or thoracotomy, n (%) | 232 (13.3) | 553 (3.1) | 38.1 |
| Early neurosurgical intervention, n (%) | 102 (5.9) | 289 (1.6) | 22.6 |
| ^a^ Standardized differences ≥10% represent meaningful differences between groups  ^b^ Presenting systolic blood pressure ≤ 90 mmHg  ^c^ Transfusion of packed red blood cells within 12 hours of arrival  ^d^ Procedure performed within 48 hours of arrival | | | |
