# Supplementary material for: Timing of femoral shaft fracture fixation following major trauma: A retrospective cohort study of United States trauma centers
Source: PLoS Med. 2017 Jul 5;14(7):e1002336. doi: 10.1371/journal.pmed.1002336 (PMC5497944; doi:10.1371/journal.pmed.1002336)
Supplement: S3 Table — (DOCX) [file pmed.1002336.s003.docx]

| **Supplementary Table 3.** Point estimates and 95% confidence intervals of center-level random effects for delayed fixation | | | |
| --- | --- | --- | --- |
| **Center** | **OR** | **Lower Confidence Limit** | **Upper Confidence Limit** |
| **1** | 0.17 | 0.09 | 0.30 |
| **2** | 0.17 | 0.09 | 0.32 |
| **3** | 0.18 | 0.11 | 0.31 |
| **4** | 0.24 | 0.11 | 0.50 |
| **5** | 0.26 | 0.16 | 0.43 |
| **6** | 0.31 | 0.12 | 0.77 |
| **7** | 0.31 | 0.19 | 0.50 |
| **8** | 0.33 | 0.13 | 0.83 |
| **9** | 0.34 | 0.20 | 0.59 |
| **10** | 0.35 | 0.15 | 0.82 |
| **11** | 0.35 | 0.21 | 0.58 |
| **12** | 0.38 | 0.19 | 0.75 |
| **13** | 0.38 | 0.21 | 0.68 |
| **14** | 0.41 | 0.25 | 0.68 |
| **15** | 0.41 | 0.20 | 0.84 |
| **16** | 0.42 | 0.27 | 0.66 |
| **17** | 0.47 | 0.24 | 0.92 |
| **18** | 0.48 | 0.29 | 0.79 |
| **19** | 0.49 | 0.23 | 1.05 |
| **20** | 0.49 | 0.29 | 0.83 |
| **21** | 0.51 | 0.20 | 1.33 |
| **22** | 0.52 | 0.28 | 0.95 |
| **23** | 0.53 | 0.26 | 1.09 |
| **24** | 0.54 | 0.27 | 1.05 |
| **25** | 0.55 | 0.38 | 0.80 |
| **26** | 0.55 | 0.29 | 1.06 |
| **27** | 0.56 | 0.30 | 1.06 |
| **28** | 0.56 | 0.36 | 0.86 |
| **29** | 0.56 | 0.26 | 1.20 |
| **30** | 0.57 | 0.32 | 0.99 |
| **31** | 0.57 | 0.37 | 0.90 |
| **32** | 0.57 | 0.31 | 1.05 |
| **33** | 0.58 | 0.40 | 0.83 |
| **34** | 0.58 | 0.31 | 1.08 |
| **35** | 0.58 | 0.33 | 1.04 |
| **36** | 0.59 | 0.36 | 0.97 |
| **37** | 0.60 | 0.33 | 1.12 |
| **38** | 0.61 | 0.34 | 1.11 |
| **39** | 0.61 | 0.34 | 1.11 |
| **40** | 0.62 | 0.25 | 1.55 |
| **41** | 0.62 | 0.35 | 1.10 |
| **42** | 0.63 | 0.38 | 1.04 |
| **43** | 0.64 | 0.36 | 1.11 |
| **44** | 0.64 | 0.43 | 0.94 |
| **45** | 0.64 | 0.29 | 1.40 |
| **46** | 0.65 | 0.28 | 1.53 |
| **47** | 0.65 | 0.31 | 1.38 |
| **48** | 0.66 | 0.38 | 1.14 |
| **49** | 0.68 | 0.42 | 1.09 |
| **50** | 0.69 | 0.37 | 1.28 |
| **51** | 0.69 | 0.33 | 1.44 |
| **52** | 0.70 | 0.45 | 1.09 |
| **53** | 0.70 | 0.40 | 1.23 |
| **54** | 0.71 | 0.36 | 1.40 |
| **55** | 0.71 | 0.52 | 0.96 |
| **56** | 0.71 | 0.37 | 1.39 |
| **57** | 0.72 | 0.46 | 1.15 |
| **58** | 0.73 | 0.38 | 1.40 |
| **59** | 0.74 | 0.43 | 1.27 |
| **60** | 0.75 | 0.47 | 1.21 |
| **61** | 0.76 | 0.39 | 1.51 |
| **62** | 0.77 | 0.41 | 1.43 |
| **63** | 0.77 | 0.56 | 1.05 |
| **64** | 0.77 | 0.46 | 1.28 |
| **65** | 0.77 | 0.48 | 1.24 |
| **66** | 0.79 | 0.42 | 1.48 |
| **67** | 0.79 | 0.36 | 1.73 |
| **68** | 0.79 | 0.37 | 1.68 |
| **69** | 0.80 | 0.43 | 1.50 |
| **70** | 0.81 | 0.51 | 1.29 |
| **71** | 0.81 | 0.53 | 1.24 |
| **72** | 0.82 | 0.50 | 1.33 |
| **73** | 0.82 | 0.55 | 1.23 |
| **74** | 0.84 | 0.57 | 1.24 |
| **75** | 0.84 | 0.50 | 1.42 |
| **76** | 0.84 | 0.39 | 1.81 |
| **77** | 0.85 | 0.39 | 1.84 |
| **78** | 0.85 | 0.52 | 1.40 |
| **79** | 0.85 | 0.45 | 1.61 |
| **80** | 0.86 | 0.45 | 1.61 |
| **81** | 0.86 | 0.54 | 1.37 |
| **82** | 0.86 | 0.56 | 1.34 |
| **83** | 0.87 | 0.52 | 1.45 |
| **84** | 0.87 | 0.50 | 1.51 |
| **85** | 0.88 | 0.56 | 1.38 |
| **86** | 0.89 | 0.56 | 1.40 |
| **87** | 0.89 | 0.40 | 1.97 |
| **88** | 0.89 | 0.46 | 1.72 |
| **89** | 0.90 | 0.52 | 1.56 |
| **90** | 0.91 | 0.57 | 1.43 |
| **91** | 0.91 | 0.50 | 1.63 |
| **92** | 0.91 | 0.46 | 1.83 |
| **93** | 0.92 | 0.60 | 1.40 |
| **94** | 0.94 | 0.48 | 1.86 |
| **95** | 0.95 | 0.55 | 1.63 |
| **96** | 0.95 | 0.47 | 1.93 |
| **97** | 0.97 | 0.68 | 1.38 |
| **98** | 0.97 | 0.53 | 1.77 |
| **99** | 0.98 | 0.51 | 1.87 |
| **100** | 0.98 | 0.46 | 2.11 |
| **101** | 0.98 | 0.66 | 1.46 |
| **102** | 1.00 | 0.42 | 2.38 |
| **103** | 1.00 | 0.63 | 1.57 |
| **104** | 1.00 | 0.39 | 2.58 |
| **105** | 1.01 | 0.54 | 1.87 |
| **106** | 1.01 | 0.79 | 1.30 |
| **107** | 1.01 | 0.49 | 2.08 |
| **108** | 1.02 | 0.58 | 1.79 |
| **109** | 1.03 | 0.59 | 1.78 |
| **110** | 1.03 | 0.36 | 2.92 |
| **111** | 1.06 | 0.76 | 1.49 |
| **112** | 1.07 | 0.55 | 2.06 |
| **113** | 1.08 | 0.58 | 2.03 |
| **114** | 1.08 | 0.76 | 1.55 |
| **115** | 1.09 | 0.57 | 2.09 |
| **116** | 1.09 | 0.54 | 2.23 |
| **117** | 1.10 | 0.75 | 1.61 |
| **118** | 1.10 | 0.75 | 1.62 |
| **119** | 1.11 | 0.61 | 2.00 |
| **120** | 1.13 | 0.69 | 1.86 |
| **121** | 1.13 | 0.83 | 1.54 |
| **122** | 1.14 | 0.67 | 1.92 |
| **123** | 1.14 | 0.76 | 1.73 |
| **124** | 1.15 | 0.51 | 2.60 |
| **125** | 1.16 | 0.64 | 2.11 |
| **126** | 1.16 | 0.63 | 2.13 |
| **127** | 1.16 | 0.71 | 1.89 |
| **128** | 1.16 | 0.72 | 1.87 |
| **129** | 1.17 | 0.54 | 2.52 |
| **130** | 1.17 | 0.76 | 1.79 |
| **131** | 1.18 | 0.48 | 2.89 |
| **132** | 1.18 | 0.80 | 1.75 |
| **133** | 1.19 | 0.83 | 1.71 |
| **134** | 1.20 | 0.70 | 2.04 |
| **135** | 1.20 | 0.63 | 2.26 |
| **136** | 1.20 | 0.76 | 1.89 |
| **137** | 1.21 | 0.76 | 1.92 |
| **138** | 1.21 | 0.67 | 2.18 |
| **139** | 1.21 | 0.55 | 2.67 |
| **140** | 1.23 | 0.62 | 2.41 |
| **141** | 1.24 | 0.75 | 2.04 |
| **142** | 1.24 | 0.71 | 2.17 |
| **143** | 1.24 | 0.59 | 2.61 |
| **144** | 1.25 | 0.81 | 1.92 |
| **145** | 1.26 | 0.68 | 2.34 |
| **146** | 1.26 | 0.76 | 2.09 |
| **147** | 1.27 | 0.69 | 2.36 |
| **148** | 1.28 | 0.73 | 2.26 |
| **149** | 1.29 | 0.76 | 2.18 |
| **150** | 1.30 | 0.97 | 1.75 |
| **151** | 1.31 | 0.67 | 2.57 |
| **152** | 1.32 | 0.60 | 2.88 |
| **153** | 1.32 | 0.73 | 2.40 |
| **154** | 1.33 | 0.91 | 1.92 |
| **155** | 1.33 | 0.84 | 2.09 |
| **156** | 1.36 | 0.79 | 2.32 |
| **157** | 1.37 | 0.79 | 2.36 |
| **158** | 1.37 | 0.80 | 2.35 |
| **159** | 1.37 | 0.86 | 2.20 |
| **160** | 1.39 | 0.66 | 2.93 |
| **161** | 1.39 | 0.77 | 2.53 |
| **162** | 1.40 | 0.91 | 2.15 |
| **163** | 1.41 | 0.80 | 2.48 |
| **164** | 1.43 | 0.82 | 2.47 |
| **165** | 1.43 | 0.94 | 2.18 |
| **166** | 1.43 | 0.87 | 2.37 |
| **167** | 1.44 | 0.78 | 2.63 |
| **168** | 1.44 | 1.00 | 2.08 |
| **169** | 1.44 | 0.79 | 2.64 |
| **170** | 1.45 | 0.90 | 2.35 |
| **171** | 1.47 | 0.57 | 3.78 |
| **172** | 1.48 | 0.76 | 2.90 |
| **173** | 1.49 | 0.92 | 2.41 |
| **174** | 1.50 | 0.97 | 2.31 |
| **175** | 1.52 | 0.84 | 2.75 |
| **176** | 1.54 | 0.89 | 2.69 |
| **177** | 1.57 | 0.87 | 2.83 |
| **178** | 1.59 | 0.88 | 2.88 |
| **179** | 1.59 | 0.67 | 3.78 |
| **180** | 1.61 | 1.09 | 2.38 |
| **181** | 1.62 | 0.76 | 3.46 |
| **182** | 1.66 | 1.10 | 2.51 |
| **183** | 1.66 | 0.65 | 4.28 |
| **184** | 1.67 | 0.71 | 3.95 |
| **185** | 1.67 | 0.90 | 3.11 |
| **186** | 1.70 | 0.99 | 2.89 |
| **187** | 1.75 | 0.87 | 3.50 |
| **188** | 1.76 | 1.06 | 2.91 |
| **189** | 1.86 | 0.99 | 3.50 |
| **190** | 1.87 | 1.20 | 2.91 |
| **191** | 1.87 | 0.79 | 4.42 |
| **192** | 1.93 | 1.20 | 3.10 |
| **193** | 1.93 | 1.15 | 3.23 |
| **194** | 2.02 | 1.52 | 2.68 |
| **195** | 2.02 | 1.22 | 3.34 |
| **196** | 2.09 | 1.47 | 2.96 |
| **197** | 2.12 | 1.16 | 3.87 |
| **198** | 2.21 | 1.52 | 3.22 |
| **199** | 2.21 | 1.47 | 3.33 |
| **200** | 2.24 | 1.67 | 3.00 |
| **201** | 2.25 | 1.22 | 4.16 |
| **202** | 2.30 | 1.41 | 3.74 |
| **203** | 2.32 | 1.21 | 4.47 |
| **204** | 2.40 | 1.45 | 3.97 |
| **205** | 2.42 | 1.53 | 3.82 |
| **206** | 2.51 | 1.02 | 6.17 |
| **207** | 2.65 | 2.03 | 3.47 |
| **208** | 2.73 | 1.52 | 4.90 |
| **209** | 2.80 | 1.99 | 3.95 |
| **210** | 2.93 | 1.90 | 4.51 |
| **211** | 3.05 | 1.75 | 5.34 |
| **212** | 3.33 | 1.52 | 7.31 |
| **213** | 3.57 | 1.60 | 7.96 |
| **214** | 3.60 | 1.69 | 7.68 |
| **215** | 4.24 | 2.45 | 7.36 |
| **216** | 4.66 | 2.79 | 7.77 |
