# Supplementary material for: Timing of femoral shaft fracture fixation following major trauma: A retrospective cohort study of United States trauma centers
Source: PLoS Med. 2017 Jul 5;14(7):e1002336. doi: 10.1371/journal.pmed.1002336 (PMC5497944; doi:10.1371/journal.pmed.1002336)
Supplement: S4 Table — (DOCX) [file pmed.1002336.s004.docx]

| **Supplementary Table 4.** Cumulative proportion of patients with fixation over time from ED arrival | | | |
| --- | --- | --- | --- |
| **Quartile 1: Lowest quartile of delayed fixation** | | | |
| Total N = 4,779 | | | |
| Hours from ED Arrival | Fixation, N | % | Cumulative % |
| 4 | 887 | 18.6 | 18.6 |
| 8 | 1110 | 23.2 | 41.8 |
| 12 | 636 | 13.3 | 55.1 |
| 16 | 584 | 12.2 | 67.3 |
| 20 | 540 | 11.3 | 78.6 |
| 24 | 376 | 7.9 | 86.5 |
| 28 | 144 | 3.0 | 89.5 |
| 32 | 80 | 1.7 | 91.2 |
| 36 | 48 | 1.0 | 92.2 |
| 40 | 56 | 1.2 | 93.3 |
| 44 | 51 | 1.1 | 94.4 |
| 48 | 46 | 1.0 | 95.4 |
| 52 | 25 | 0.5 | 95.9 |
| 56 | 15 | 0.3 | 96.2 |
| 60 | 16 | 0.3 | 96.5 |
| 64 | 14 | 0.3 | 96.8 |
| 68 | 15 | 0.3 | 97.2 |
| 72 | 12 | 0.3 | 97.4 |
|  |  |  |  |
| **Quartile 2** | | | |
| Total N = 4,897 | | | |
| Hours from ED Arrival | Fixation, N | % | Cumulative % |
| 4 | 404 | 8.2 | 8.2 |
| 8 | 869 | 17.7 | 26.0 |
| 12 | 650 | 13.3 | 39.3 |
| 16 | 720 | 14.7 | 54.0 |
| 20 | 675 | 13.8 | 67.8 |
| 24 | 483 | 9.9 | 77.6 |
| 28 | 247 | 5.0 | 82.7 |
| 32 | 145 | 3.0 | 85.6 |
| 36 | 90 | 1.8 | 87.5 |
| 40 | 90 | 1.8 | 89.3 |
| 44 | 73 | 1.5 | 90.8 |
| 48 | 64 | 1.3 | 92.1 |
| 52 | 48 | 1.0 | 93.1 |
| 56 | 18 | 0.4 | 93.4 |
| 60 | 23 | 0.5 | 93.9 |
| 64 | 28 | 0.6 | 94.5 |
| 68 | 23 | 0.5 | 95.0 |
| 72 | 22 | 0.4 | 95.4 |
|  |  |  |  |
| **Quartile 3** | | | |
| Total N = 4,293 | | | |
| Hours from ED Arrival | Fixation, N | % | Cumulative % |
| 4 | 340 | 7.9 | 7.9 |
| 8 | 668 | 15.6 | 23.5 |
| 12 | 483 | 11.3 | 34.7 |
| 16 | 539 | 12.6 | 47.3 |
| 20 | 570 | 13.3 | 60.6 |
| 24 | 418 | 9.7 | 70.3 |
| 28 | 283 | 6.6 | 76.9 |
| 32 | 140 | 3.3 | 80.2 |
| 36 | 97 | 2.3 | 82.4 |
| 40 | 99 | 2.3 | 84.7 |
| 44 | 109 | 2.5 | 87.3 |
| 48 | 79 | 1.8 | 89.1 |
| 52 | 46 | 1.1 | 90.2 |
| 56 | 38 | 0.9 | 91.1 |
| 60 | 22 | 0.5 | 91.6 |
| 64 | 40 | 0.9 | 92.5 |
| 68 | 34 | 0.8 | 93.3 |
| 72 | 37 | 0.9 | 94.2 |
|  |  |  |  |
| **Quartile 4: Highest quartile of delayed fixation** | | | |
| Total N = 4,024 | | | |
| Hours from ED Arrival | Fixation, N | % | Cumulative % |
| 4 | 184 | 4.6 | 4.6 |
| 8 | 530 | 13.2 | 17.7 |
| 12 | 400 | 9.9 | 27.7 |
| 16 | 440 | 10.9 | 38.6 |
| 20 | 487 | 12.1 | 50.7 |
| 24 | 373 | 9.3 | 60.0 |
| 28 | 280 | 7.0 | 66.9 |
| 32 | 178 | 4.4 | 71.4 |
| 36 | 124 | 3.1 | 74.5 |
| 40 | 145 | 3.6 | 78.1 |
| 44 | 117 | 2.9 | 81.0 |
| 48 | 98 | 2.4 | 83.4 |
| 52 | 76 | 1.9 | 85.3 |
| 56 | 49 | 1.2 | 86.5 |
| 60 | 38 | 0.9 | 87.5 |
| 64 | 45 | 1.1 | 88.6 |
| 68 | 53 | 1.3 | 89.9 |
| 72 | 45 | 1.1 | 91.0 |
