# Supplementary material for: Timing of femoral shaft fracture fixation following major trauma: A retrospective cohort study of United States trauma centers
Source: PLoS Med. 2017 Jul 5;14(7):e1002336. doi: 10.1371/journal.pmed.1002336 (PMC5497944; doi:10.1371/journal.pmed.1002336)
Supplement: S1 Study Protocol — (DOCX) [file pmed.1002336.s006.docx]

**Protocol version 3: January 12, 2016**

**Exploring variability in timing of fracture fixation across TQIP centers**

James P Byrne MD^1,2^, Avery B Nathens MD PhD^1,2^, David Gomez MD PhD^1,2^, Richard Jenkinson MD^2,3^,

1. Department of Surgery, Division of General Surgery, University of Toronto, ON, Canada
2. Sunnybrook Research Institute, Sunnybrook Health Sciences Centre, Toronto, ON, Canada
3. Department of Surgery, Division of Orthopedic Surgery, University of Toronto, ON, Canada

**Note on Changes to Protocol:**

We had initially planned to perform a patient-level analysis using a propensity matching methodology to measure the association between delayed fixation and granular patient-level outcomes. However, because several previous studies have examined the influence of timing of fixation on patient outcomes (as highlighted in the introduction to our paper), we felt this would not add value. It also would have made the manuscript more confusing to have several disparate analyses. For this reason, we chose a more focused approach, to examine factors influencing delayed fixation at the patient-level, hospital variability, and the association with outcomes at the hospital-level.

**Background**

The Trauma Quality Improvement Program (TQIP) of the American College of Surgeons (ACS) Committee on Trauma aims to improve trauma center performance through the evaluation of the relationships between processes and outcomes. Through the use of standardized data collection and risk-adjusted benchmarking, TQIP explores variability in trauma center performance in order to identify high and low performing centers.

One proposed measure of trauma center performance has been the timing of fixation for mid-shaft femur fractures. However, significant variability in timing of fracture fixation has been identified. The reasons behind this variability, and the impact on clinical outcomes, remains unclear.

**Methods**

***Study Design***

This is a retrospective cohort study in which the primary objective is to evaluate the relationship between the timing of fracture fixation for mid-shaft femur fractures, open tibial shaft fractures and hip fractures in order to better understand the barriers behind delays in fracture fixation.

***Data Source***

TQIP is run by the ACS Committee on Trauma. It collects patient-level and center-level data on Level I or II ACS verified or state designated trauma centers. Baseline and injury characteristics of injured adult (age ≥ 16 years) patients with at least one valid trauma International Classification of Disease version 9 (ICD-9) code in the range of 800–959.9, and at least one Abbreviated Injury Scale (AIS) ≥ 3 in body regions 1 – 8 are captured. Patients with the following ICD-9 codes: late effects (905-909.9), superficial injuries (910-924.9), and foreign bodies (930-939.9) are excluded.

***Inclusion/Exclusion Criteria***

We will include patients who received care during the year 2012 – 2015 in any level I or level II trauma center pariticpating in TQIP. We will identify adult patients (age> 16 years), with and Injury Severity Score =>9 and a blunt mechanical mechanism of injury.

The cohort of interest will be identified using the ICD-9 codes for midshaft femur fracture (821.01, 821.11). The cohort will be further limited to patients who underwent a surgical fixation procedure of the femur fracture, identified by ICD-9 codes for ORIF (79.35), CRIF (79.15), Fixation without reduction (78.55), and External Fixation (78.15).

***Analytic Approach***

Our approach will comprise of the following patient and hospital-level analyses.

Patient-level Analyses

1. Predictors of delayed fixation (>24 hours) will be identified using hierarchical logistic regression, accounting for clustering at the hospital-level
2. Propensity score matching will be used to match patients who underwent early fixation (EF, ≤24 hours), to those who underwent (DF, >24 hours), on patient baseline and injury characteristics. Hierarchical logistic regression will be used to estimate the odds of important clinical outcomes in patients who received DF vs. EF, accounting for the paired nature of the data and clustering in hospitals. Important clinical outcomes will include PE, DVT, ARDS, Pneumonia, Death, as well as Hospital LOS.

Hospital-level Analyses

1. Variability between hospitals in delayed fixation will be quantified using hierarchical logistic regression. Specifically, hospital outlier status for performing delayed fixation will be examined. The median odds ratio (MOR) will be used to quantify variance at the hospital-level, after adjusting for patient-level covariates.
2. The impact of hospital practices with respect to delayed fixation on clinical outcomes will be examined. Specifically, the random effects estimates of hospital odds of performing delayed fixation will be used to rank trauma centers on propensity for delayed fixation. Rates of PE, DVT, ARDS, Pneumonia, Death, as well as Hospital LOS will be compared across quartiles of tendency for delayed fixation.
